# Study on the Preparation and Compressive Strength of Boron Mud-Based Basic Magnesium Sulfate Cement

**DOI:** 10.3390/ma17133301

**Published:** 2024-07-04

**Authors:** Jiankun Li, Xiaowei Gu, Shenyu Wang, Zhihang Hu, Ziyang Hu, Xiaqing Li

**Affiliations:** 1 Science and Technology Innovation Center of Smart Water and Resource Environment, Northeastern University, Shenyang 110819, China; 2 Liaoning Institute of Technological Innovation in Solid Waste Utilization, Shenyang 110819, China

**Keywords:** boron mud, basic magnesium sulfate cement, 5·1·7 phase, compressive strength, solid waste treatment

## Abstract

The direct discharge of boron mud poses significant environmental hazards to soil and groundwater. Despite extensive research efforts, the reprocessing of boron mud has not yielded significant advancements. Recently, the development of magnesium cement has spurred interest in the reutilization of boron mud. However, the direct treatment of boron mud remains challenging, necessitating pre-treatment in most studies to achieve substantial results. Consequently, research on the direct incorporation of untreated boron mud is scarce. This study explores the feasibility of using uncalcined boron mud as a base material in basic magnesium sulfate cement (BMSC), composed of lightly calcined magnesia and magnesium sulfate heptahydrate. The effects of varying boron mud content on the compressive strength of the BMSC system were investigated. The results indicate that the 5·1·7 phase is the primary strength phase of BMSC. When the boron mud content is 30%, the uncalcined boron mud has a minimal impact on the formation of the 5·1·7 phase. Additionally, the 28 days compressive strength of BMSC-B30 showed a slight difference compared to the control group BMSC-C, registering at 66.7 MPa. TG-DSC analysis revealed that the presence of a small amount of boron mud inhibits the micro-expansion trend of the BMSC structure. Furthermore, XRD and SEM analyses confirmed that the addition of uncalcined boron mud does not significantly alter the phase structure of the 5·1·7 phase in BMSC. This study provides a foundational basis for the long-term development of direct boron mud treatment.

## 1. Introduction

Boron mud is a solid waste produced during the production of borax. It occupies a large amount of land and causes groundwater pollution [1,2]. Its primary mineral components include forsterite (Mg_2_SiO_4_), magnesite (MgCO_3_), and quartz (SiO_2_) [3]. Additionally, moisture loss from the stacking process can lead to dust dispersion and severe air pollution [4]. Approximately 3–4 tons of boron mud are generated for every ton of borax produced [5]. Currently, many attempts focus on recovering MgO and SiO_2_ from boron mud due to its high content of these components. Efforts to extract more magnesium and silicon from boron mud and to prepare related magnesium compound products have been documented. Yan, B.X., et al. observed in their extraction experiments that the concentrations of Mg, B, Na, and other elements in the extract were higher than those in control soil extracts. High boron content leads to soil salinization, hindering crop growth [6,7]. Ning, Z., et al. used waste sulfuric acid to produce over 90% MgSO_4_·7H_2_O from boron mud, although the cost was high [8]. Ning, Z., et al. studied the potential application of boron mud in cement, finding that its incorporation can increase the hydration rate of cement but significantly decrease its later compressive strength [9].

Basic magnesium sulfate cement (BMSC) is a new type of refractory building material developed after magnesium phosphate cement (MPC) and magnesium oxychloride cement (MOC) [10]. Consequently, BMSC has been widely used in the production of various fireproof and decorative wall panels [11]. Wu, C.Y., et al. innovatively incorporated additives into existing magnesium oxysulfate cement (MOS) to obtain a new type of cement with excellent performance [12]. The MgO-MgSO_4_-H_2_O ternary cementitious system, composed of active MgO and magnesium sulfate heptahydrate solution, derives its strength from the phase 5Mg(OH)_2_·MgSO_4_·7H_2_O (phase 5·1·7) [13]. Moreover, BMSC is an air-hardening cement with rapid hardening [14], excellent fire resistance, low thermal conductivity, high stability, and good chemical resistance [15]. The hydration product, primarily the 5·1·7 phase, enhances the mechanical properties of BMSC [16].

Currently, research on the utilization of boron resources is increasing globally [17], and the pollution problem of boron tailings is receiving more attention [18]. To address this issue, some scholars focus on producing building materials to consume and reuse excess boron tailings powder. However, the low reactivity of boron mud powder limits its utilization in the construction field. Some studies have attempted to add boron mud to cement systems, requiring the pretreatment of the boron mud. The most common method used is thermal activation [19]. For example, Yu, J., et al. used calcined boron mud instead of dead-burned MgO to prepare magnesium phosphate cement (MPC), resulting in cement with sufficient setting time, good fluidity, and high compressive strength [20]. Kavas, T., et al. in 2015 studied the feasibility of producing dolomite cement using red mud and boron mud. The experimental results showed that even after calcining boron mud at 1300 °C, the produced cement had a strength of only 22.5 MPa [21]. Additionally, Liang et al. in 2011 explored the addition of different alcohol-based modifiers to a mixture of magnesium oxalate cement and boron mud. The study indicated that the low alkalinity of the mixture was beneficial for the conventional curing of the cement [22].

The technology of BMSC is becoming more mature, and this type of cement is renowned for its high pressure and flexural strength [23]. The BMSC system mainly relies on the activity of lightly burned magnesia to improve the overall mechanical properties of the cement [24]. The activity of boron mud can only obtain a small amount of active magnesia through thermal activation, which is energy intensive. The hydration method measured only 10% active magnesia in the calcined magnesia. Zhang, X., et al.’s analysis showed that incorporating calcined boron mud into BMSC did not significantly improve its performance and did not promote the formation of the 5·1·7 phase [25]. Moreover, in the practical application of BMSC, lightly burned magnesia powder usually accounts for more than 50% of the total dry raw material weight, with over 60% of the MgO in BMSC not participating in the hydration reaction, serving only as a filler [26]. Therefore, some potentially reactive alternative materials can replace part of the lightly burned magnesia powder, such as silica fume, fly ash, red mud, metakaolin, carbide slag, and desulfurized gypsum [27]. Boron mud is also a solid waste among these materials. In related studies, the incorporation of solid waste generally does not exceed 40% [28]. Although some materials participate in the reaction in BMSC, their low activity still limits their effectiveness [29]. Replacing lightly burned magnesia powder can also achieve cost reduction.

This study attempts to prepare BMSC by directly replacing lightly burned magnesia with boron mud, adopting a direct consumption method without pretreatment. Currently, there is no research on using boron mud directly to replace magnesia raw materials in BMSC. Compared to the pretreatment method, directly incorporating boron mud can save production costs. Additionally, due to the intense reaction during the early hydration process of BMSC, leading to cracking [30], the low reactivity of boron mud may mitigate the early hydration reaction rate of BMSC.

In this study, we aim to prepare BMSC by replacing lightly burned magnesia with uncalcined boron mud. First, the properties of boron mud powder before and after calcination were studied. The differences between uncalcined and pretreated boron mud were explored. The compressive strength and hydration products of BMSC prepared by replacing magnesia raw materials with uncalcined boron mud were investigated. The study examined whether boron mud could improve the performance of BMSC and its effect on the formation of the 5·1·7 phase. Using comprehensive analysis methods such as XRD, TG-DSC curve analysis, FTIR analysis, and SEM imaging, the optimal mix ratio of BMSC was studied. The experimental results are expected to provide a basis for the long-term development of solid waste resources with the properties of boron mud.

## 2. Experimental

### 2.1. Materials

**Figure 1:**
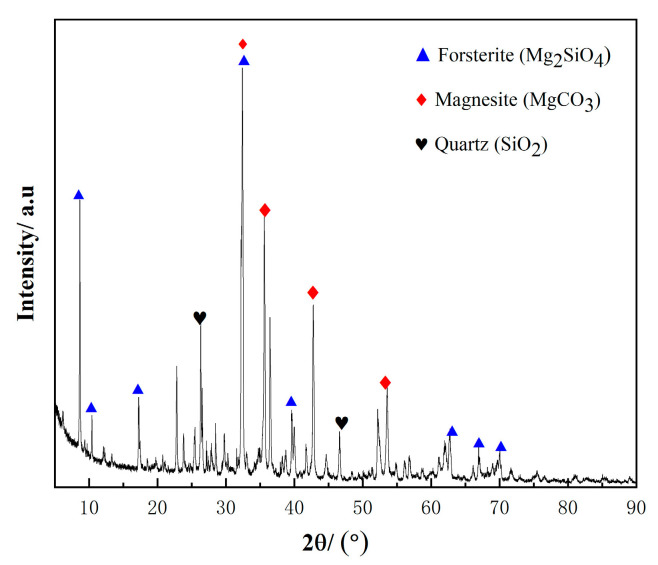
XRD pattern of boron mud (forsterite (Mg_2_SiO_4_), magnesite (MgCO_3_), quartz (SiO_2_)).

**Figure 2:**
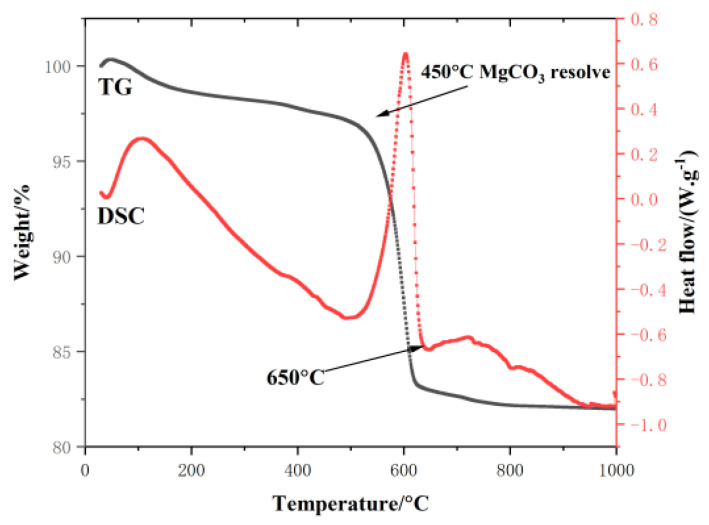
TG-DSC curve of boron mud.

The boron mud used in this experiment was obtained from Yingkou Ding’an Group (Yingkou, China), a byproduct of borax production. The particle sizes were less than 73.5 μm in 93% of the mud. The chemical composition of the boron mud is listed in Table 1, determined using a PANalytical Axios X-ray fluorescence spectrometer (XRF). The test sample was then strained through 75 μm sieve. Figure 1 shows the phase assemblages of boron mud, characterized by a SmartLab Intelligent X-ray diffractometer (XRD). Figure 2 shows the TG-DSC curve of the boron mud by simultaneous thermal analyzer, STA449F3, Netzsch, Germany. Lightly burned magnesia was provided by the Xifeng Group of Dashiqiao, China, calcined at 850 °C for 2 h. The content of active magnesia (a-MgO) was approximately 67.5%, as determined by the magnesium oxide activity detection method (as described in Section 2.2.2). The chemical composition of lightly burned magnesia is listed in Table 2, determined using a PANalytical Axios X-ray fluorescence spectrometer (XRF). Magnesium sulfate heptahydrate (MgSO_4_·7H_2_O), reagent grade with a purity of not less than 98%, was dissolved in water to prepare a 25% mass fraction magnesium sulfate solution. It was produced by the Xifeng Group of Dashiqiao, China. The chemical composition of magnesium sulfate heptahydrate crystals is listed in Table 3, determined using a PANalytical Axios X-ray fluorescence spectrometer (XRF). Deionized water from Liaoning Province was used in the experiments. Citric acid, reagent grade, compliant with the Chinese standard “Chemical reagent monohydrate Citric acid (Citric acid)” (GB/T 9855-2008) [31], was produced in Hebei Province, China. It was added to each test slurry to facilitate the formation of the 5·1·7 phase.

#### Properties of Calcined and Uncalcined Boron Mud

According to XRF analysis (as shown in Table 1), the boron mud contains 53.51% MgO and 27.88% SiO_2_. Jiang, T., et al.’s study used XRF analysis to detect only magnesium-containing oxides [32]; however, this form of MgO is not suitable for magnesium sulfate cement (BMSC). Xuijun, Fu, et al. confirmed that the main oxide components are MgCO_3_ and Mg_2_SiO_4_, with MgCO_3_ accounting for only 30% of the total MgO content [33]. The XRD pattern of the boron mud is shown in Figure 1, indicating that the primary minerals are forsterite (Mg_2_SiO_4_), magnesite (MgCO_3_), and quartz (SiO_2_). The activity of MgO in the boron mud, determined by the hydration method, is 0% (see Section 2.2.2), indicating that there is no active MgO in the raw boron mud. Yun Yun Yin suggested that high-temperature calcination can decompose MgCO_3_ in boron mud into MgO and CO_2_, providing a theoretical basis for preparing MgO from boron mud [34]. Figure 2 shows the TG-DSC curve of the boron mud. The weight loss rate of the boron mud stabilizes at 700 °C, indicating the complete decomposition of MgCO_3_. Above 700 °C, the weight loss rate remains relatively constant, with MgO activity initially increasing and then decreasing. However, the activity of calcined MgO determined by the hydration method is only 10%. In contrast, the MgO activity in lightly burned magnesia used in BMSC can reach 67.5%, and 10% MgO activity is insufficient to support the formation of the 5·1·7 phase. Therefore, this study did not adopt the calcination method. Moreover, this thermal treatment method increases the cost of solid waste processing.

### 2.2. Test and Characterization Methods

#### 2.2.1. Magnesium Oxide Activity Detection Method

The 10 g sample was mixed with 50 g H_2_O and put into the oven at 105 °C for 4 h, then the temperature was adjusted to 150 °C for 2 h until the weight was constant, and the activity of hydration was calculated according to Equation (1).

(1) $$W=\frac{W_{2}-W_{1}}{0.45\;W_{1}}\times 100\%$$

In the formula: *W*_1_ is the mass of the sample raw material, g; *W*_2_ is the mass of the sample after hydration, g; and 0.45 is the mass conversion factor increased after hydration of the sample. The hydration method was tested according to the Chinese standard “Light-fired Magnesia for Magnesite Products” (WB/T 1019-2002) [35].

#### 2.2.2. Specimen Preparation

**Figure 3:**
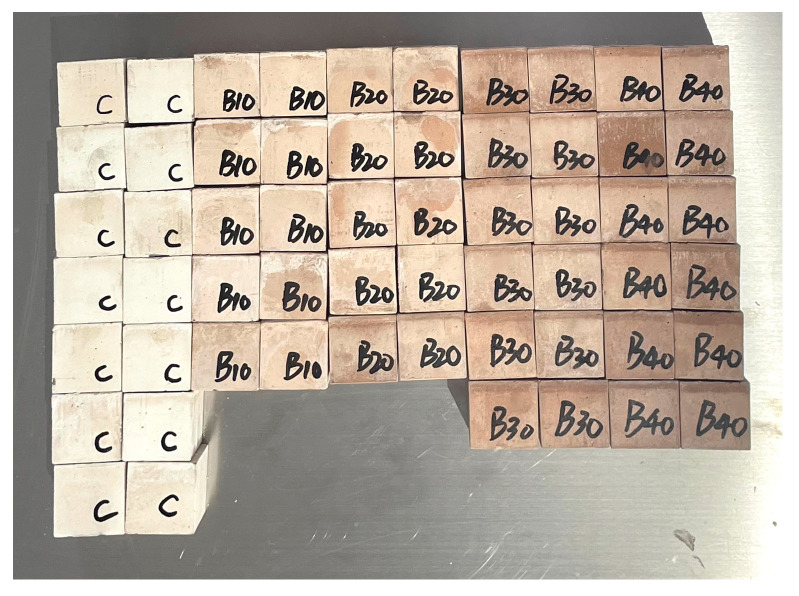
Paste specimen of BMSC samples.

**Figure 4:**
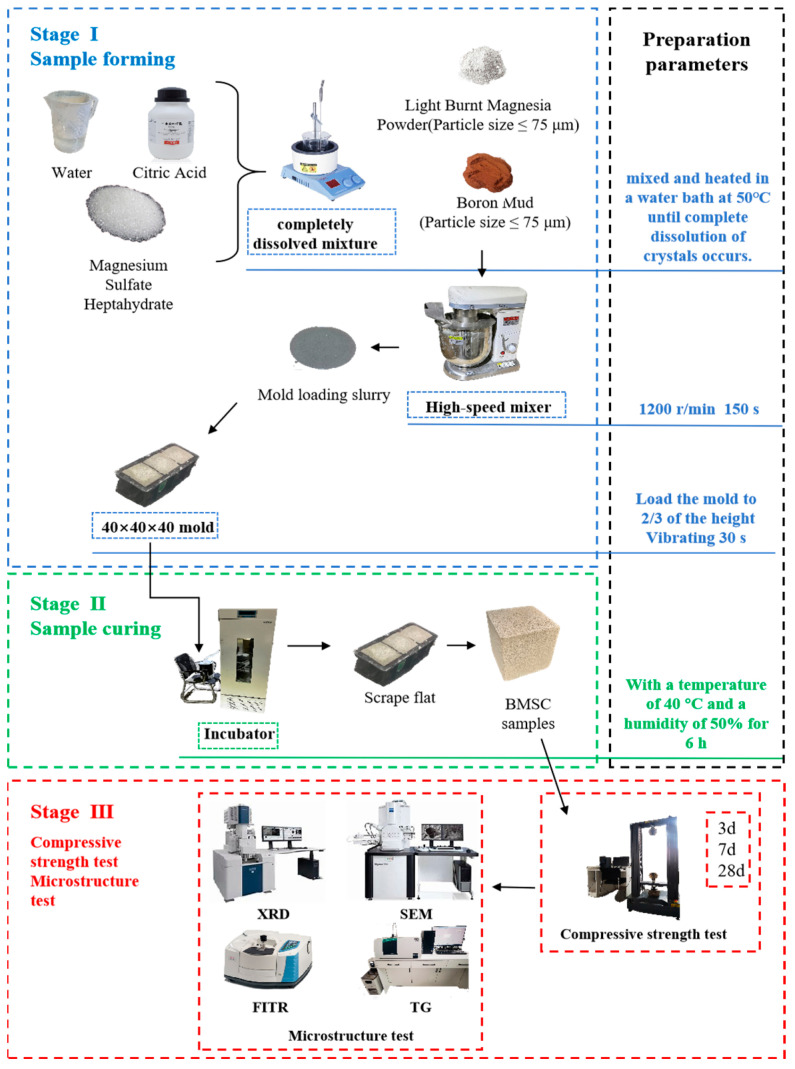
Experimental flowchart.

Due to the absence of an official national standard for the preparation of magnesium oxysulfate cement (BMSC) in China, this study was conducted with reference to the “General Portland Cement” (GB/T 175-2023) [36] standard. Additionally, we reviewed extensive previous research and conducted numerous preliminary experiments to determine the optimal mix proportion. The chosen ratio for preparing the BMSC paste was MgO:MgSO_4_:H_2_O = 100:100:1 by mass. BMSC samples were prepared using an in situ compaction procedure. First, 153.75 g of MgSO_4_·7H_2_O and 146.3 g of H_2_O were mixed and heated in a water bath at 50 °C until the crystals were fully dissolved. Then, citric acid, amounting to 1% of the mass of magnesium oxide, was added to facilitate the formation of the 5·1·7 phase. This mixture was then placed in a high-speed mixer (model NJ-160A, manufactured by Rongjid, Shanghai, China) and stirred at a low speed of 60 r/min for two minutes to prepare the magnesium sulfate paste. Next, according to the proportions listed in Table 4, a total mass of 499 g of lightly burned magnesium oxide and boron mud (with unburned boron mud mass fractions of 0%, 10%, 20%, 30%, and 40%) was added to the prepared magnesium sulfate paste. The mixture was stirred at a high speed of 300 r/min for 2 min to produce a uniform BMSC paste. This paste was then poured into steel molds with dimensions of 40 mm × 40 mm × 40 mm and vibrated on shaking table for 3 min. After curing for approximately 24 h, the samples were demolded. After that, the samples rested for 2 h to allow the surfaces to dry completely. The demolded samples were placed in a curing chamber with a relative humidity of 60 ± 5% and a temperature of 25 ± 2 °C. Figure 3 shows the paste specimen of BMSC samples. The compressive strength of the specimens was tested on the 3rd, 14th, and 28th days. the overall experimental flow is illustrated in Figure 4.

#### 2.2.3. Mechanical Properties Analysis

For the compressive strength test, a Cangzhou Longitude and Latitude 300c S electronic servo testing machine (Cangzhou, China) was employed to measure the compressive strength of 40 mm × 40 mm × 40 mm samples in accordance with the “Cement Mortar Strength Inspection Method (ISO)” (GB/T 17671-2021) [37]. The maximum load capacity of the machine during compression testing is 300 kN, with a loading speed of 2 mm/min. Compressive strength tests were conducted at 7 days, 14 days, and 28 days. Five groups with different mix ratios were tested, and three measurements were taken for each group to obtain an average value for analysis. At 28 days after various curing conditions, samples were collected and divided into smaller pieces. After being soaked in ethanol for 48 h, the samples were removed from the solution and ground using a grinding dish. Powdered samples were then sieved through a screen with a mesh size of 0.075 mm to observe changes in properties related to the formation of boron mud’s crystalline phase within the basic magnesium sulfate cement matrix under different mix ratios.

#### 2.2.4. X-ray Diffraction (XRD)

The paste specimens that had been cored were vacuum-dried at 40 °C for three days before being ground into powder and passed through a 75 μm sieve. Using a SmartLab Intelligent X-ray Diffractometer (from Malvern Instruments Limited and PANalytical B.V., Malvern, UK) with a Cu Kα X-ray source under the conditions of 40 kV and 40 mA, set λCu = 0.15418 nm, XRD was used to characterize the mineral compositions of specimens. All specimens were scanned for 10 min in the range of 5° to 90° with per step size of 0.026° (2θ). Counting time was used to determine the crystalline composition of magnesium oxysulfide cement. The 2nd (2θ) analysis was employed to identify the crystalline phases present in the cement-based samples. Each XRD measurement was conducted in triplicate to ensure the reliability and reproducibility of the results.

#### 2.2.5. Thermogravimetric Analysis (TG)

Approximately 10 mg of each cement-based sample was used for the TG analysis, and the test sample through 75 μm sieve. TG tests were performed on hardened pastes using a Netzsch STA 449 F3 simultaneous thermal analyzer under a nitrogena with constant gas flow rate of 60 mL/min atmosphere. The heating rate was 10 °C/min from 40 °C to 900 °C, and then the TG-DSC curve was recorded.

#### 2.2.6. Infrared Spectroscopy Analysis (FTIR)

As a supplement to XRD, FTIR spectra can be used to determine a material’s ability to bind with crystalline and amorphous phases, with the intensity and width of bands serving as additional indicators to determine the substance content and degree of gel polymerization. The paste specimens were pre-treated in the same way as the XRD test in Section 2.2.4, and FTIR spectra were acquired using a Nicolet iS10 FTIR spectrometer. A resolution of 4 cm^−1^ was used to test transmittance between 500 and 4000 cm^−1^.

#### 2.2.7. Microstructural and Compositional Analysis Using SEM-EDS

The scanning electron microscope-energy dispersive X-ray spectroscopy (SEM-EDS) system used in this study was manufactured by Zeiss Microscope Co., Ltd., Oberkochen, Germany. The SEM was operated at an accelerating voltage of 20 kV, with resolutions of 0.8 nm at 15 kV and 1.6 nm at 1 kV. The magnification range for the SEM was from 12× to 1,000,000×, which allowed the detailed observation of the morphology and microstructural features of the cement-based samples. For each sample type, two independent replicates were prepared and imaged to ensure consistent observations. Energy dispersive X-ray spectroscopy (EDS) was performed in conjunction with the SEM analysis to determine the elemental composition of the samples. EDS spectra were collected from multiple regions of interest within the samples. The EDS detector was calibrated using a standard reference material to ensure accuracy. Data acquisition for each spectrum was set to 60 s to achieve a sufficient signal-to-noise ratio. Elemental maps were generated to provide a spatial distribution of elements within the samples, offering comprehensive insights into their microstructural and compositional properties.

## 3. Results

### 3.1. Effect of Boron Mud Content on Cement Properties

#### 3.1.1. Analysis of BMSC Compressive Strength

**Figure 5:**
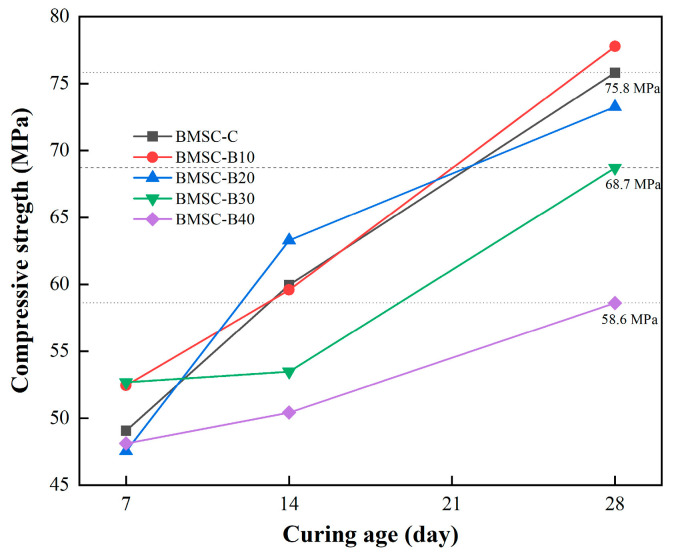
Compressive strength of BMSC sample with different mix proportions.

The compressive strength of BMSC specimens with varying mix ratios of boron mud content was measured in this study, and the results are presented in Figure 5. As the control group, BMSC-C exhibited a compressive strength of 59.96 MPa at 14 days and 75.8 MPa at 28 days. The compressive strength values at 28 days for specimens BMSC-B10, BMSC-B20, BMSC-B30, and BMSC-B40 suggested a continuous decrease in mechanical strength as the boron mud content increased. Compared to the 28-day strength value of BMSC-C, the compressive strength of BMSC-B40 might decrease to 58.6 MPa.

By comparing the compressive strength values of the five groups of specimens, it can be inferred that excessive replacement of active MgO with boron mud is likely detrimental to the enhancement of mechanical properties in boron mud-based basic magnesium sulfate cement. Sui, H.Y., et al.’s analysis suggested that this could be due to the limited content of active MgO in boron mud, where the excessive addition of boron mud as a substitute for active MgO results in insufficient availability of active MgO for reaction within the basic magnesium sulfate cement system [26]. Consequently, this may reduce the formation of the 5·1·7 phase through hydration reactions, potentially impeding the encapsulation of residual Mg_2_SiO_4_ particles and other non-reactive substances within the system, ultimately leading to a decrease in compressive strength [38]. However, it is noteworthy that BMSC-B10 exhibited higher 28-day compressive strength than BMSC-C, reaching 77.78 MPa. Ning, Z.Q., et al. explained this phenomenon, suggesting that the incorporation of uncalcined boron mud as an inert admixture, along with lightly burned magnesium oxide powder and a small quantity of magnesium compound from boron mud, might establish a state of equilibrium, thereby retarding the reaction rate of active magnesium oxide [39]. Thus, the adverse effect on compressive strength stemming from the expansion of magnesium oxide is potentially mitigated. However, the specific mechanisms require further study using more detailed molecular dynamics models.

Additionally, the 7-day compressive strength of BMSC-B10 and BMSC-B20 specimens surpassed that of BMSC-C, indicating that the incorporation of boron mud might promote early strength development in the binding material. The high early strength of BMSC-B10 could be due to the mitigation of volume expansion caused by Mg(OH)_2_. Yang, L., et al. concluded that by reducing the reaction between active magnesium oxide and H_2_O to produce Mg(OH)_2_, BMSC-B10 might mitigate volume expansion caused by Mg(OH)_2_ and ensure the dimensional stability of boron mud-based basic magnesium sulfate cement [40]. Consequently, the 28-day compressive strength of BMSC-B10 is correspondingly enhanced. Nevertheless, the long-term stability and durability of these materials under various environmental conditions need to be assessed to confirm these initial findings.

Moreover, the higher 14-day compressive strength observed in BMSC-B20 could be attributed to its ability to avoid a loose structure resulting from excessive volume expansion caused by Mg(OH)_2_ and subsequent mechanical strength reduction. Furthermore, Yang, P., et al. concluded in 2023 that the incorporation of a certain amount of boron mud might compensate for the vacancies caused by early 5·1·7 phase hydration due to its abundance in fine particles, which easily embed into the gaps resulting from micro-expansion of MgO [41]. This phenomenon might also account for the increased mid-14-day compressive strength observed in BMSC-B20.

This study primarily focuses on adding more boron mud while ensuring mechanical properties. Both BMSC-B30 and BMSC-B40 exhibit 28-day strengths exceeding 52.5 MPa; specifically, BMSC-B30 demonstrates a compressive strength of 52.68 MPa at 7 days and reaches 68.7 MPa at 28 days, meeting the standard compressive strength requirement for conventional Portland cement. Compared to other studies, the replacement amount of boron mud did not significantly affect the compressive strength due to the inherently high baseline strength of BMSC. Comparing the mechanical properties at different time points (i.e., 7 days, 14 days, and 28 days), it is evident that BMSC-B30 might exhibit greater stability in terms of strength changes compared to BMSC-B40. This stability could be crucial for practical applications, although further tests are needed to confirm the performance under long-term loading and environmental exposure.

#### 3.1.2. XRD Analysis

**Figure 6:**
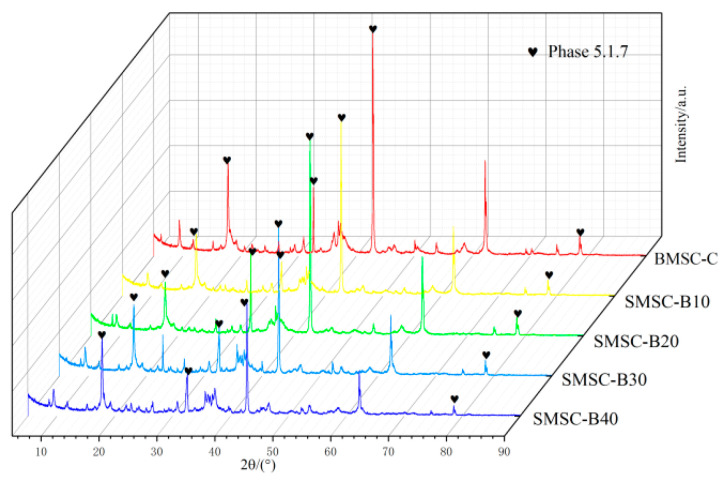
XRD of BMSC samples with different mix proportions.

**Figure 7:**
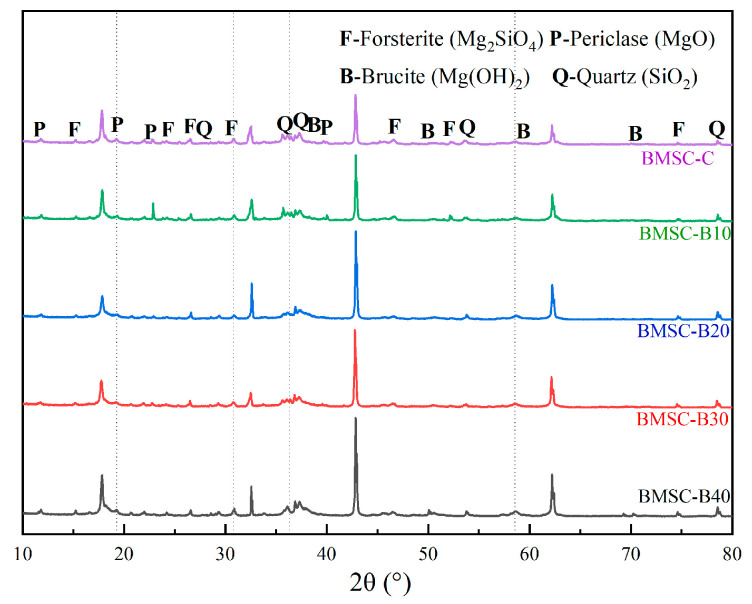
XRD analysis of other products in BMSC samples with different mix proportions (excluding the 5.1.7 Phase) (F—forsterite (Mg_2_SiO_4_); B—brucite (Mg(OH)_2_); P—periclase (MgO); Q—quartz (SiO_2_)).

To further investigate the influence of different boron mud contents on the strength of BMSC, the phase composition of BMSC specimens at a curing age of 28 days was determined using XRD analysis. The results, presented in Figure 6 and Figure 7, reveal the coexistence of multiple phases, including the predominant 5·1·7 phase, forsterite (Mg_2_SiO_4_), brucite (Mg(OH)_2_), periclase (MgO), and quartz (SiO_2_).

The XRD results in Figure 6 primarily highlight the 5·1·7 phase. The analysis of diffraction peaks corresponding to this phase in specimens BMSC-B10, BMSC-B20, BMSC-B30, and BMSC-B40 indicates that all four doping amounts resulted in the formation of the 5·1·7 phase. However, a decreasing trend in diffraction peak intensity is observed with increasing boron mud content. Since the 5·1·7 phase significantly contributes to compressive strength [42], the excessive replacement of active MgO by boron mud may lead to insufficient availability for reaction within the basic magnesium sulfate cement system, consequently reducing the generation of the hydrated 5·1·7 phase.

In addition to the 5·1·7 phase, Figure 7 reveals the presence of other microstructural phases, such as forsterite (Mg_2_SiO_4_), brucite (Mg(OH)_2_), periclase (MgO), and quartz (SiO_2_). These phases coexist with the 5·1·7 phase and influence its formation. Miao, M., et al.’s study found that an increase in surface defects of magnesium oxide, due to high activity, leads to more difficulty in forming the 5·1·7 phase because these defects can adsorb more modifier molecules [43]. However, the addition of boron mud significantly reduces the concentration of these modifier molecules, leading to a relative loss of their role and making the 5·1·7 phase harder to generate.

Despite the gradual decrease in the diffraction peak intensity of the 5·1·7 phase with increasing boron mud content, the 28-day compressive strength results for BMSC-B10, BMSC-B20, BMSC-B30, and BMSC-B40 show that these mixtures still meet the standard compressive strength of 52.5 MPa for conventional cement. This indicates that incorporating a certain amount of boron mud into the magnesium sulfate cement system does not alter the dominance of the 5·1·7 phase as the primary mechanical phase.

**Figure 8:**
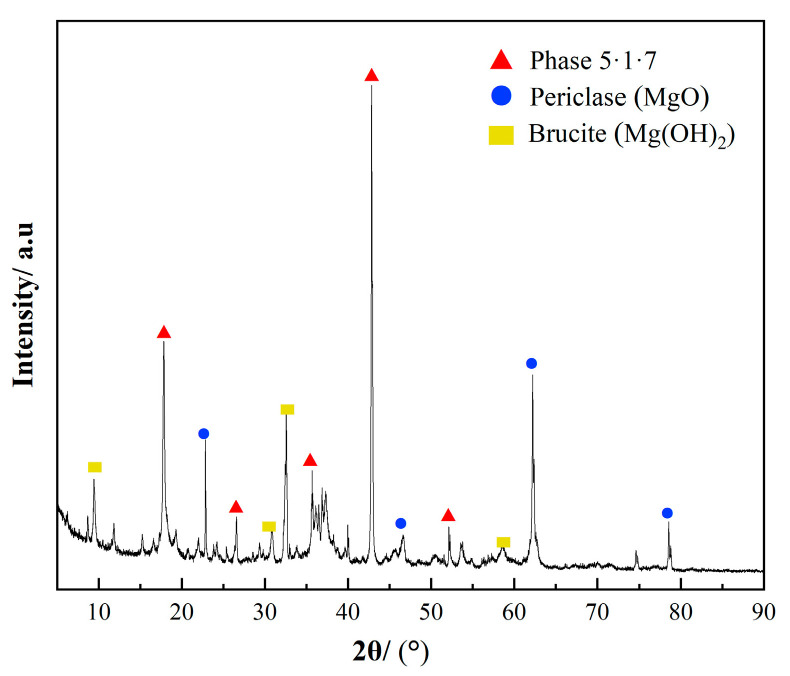
XRD pattern of BMSC-B30 sample (periclase (MgO); brucite (Mg(OH)_2_)).

The XRD spectrum of BMSC-B30 samples prepared under optimal conditions, presented in Figure 8, predominantly exhibits the 5·1·7 phase, along with minor amounts of periclase and magnesite. In contrast to other related research, whether the boron mud was calcined or not did not significantly impact the performance of BMSC; even at a 30% replacement rate, the formation of the 5·1·7 phase was ensured. However, the addition of boron mud affects the overall reaction of the BMSC paste. Some MgO does not participate in the hydration process, and the excess unreacted MgO may combine with H_2_O molecules from the air to form brucite (Mg(OH)_2_). Therefore, future research should focus on how to consistently promote the participation of MgO in the formation of the 5·1·7 phase.

#### 3.1.3. TG Analysis

**Figure 9:**
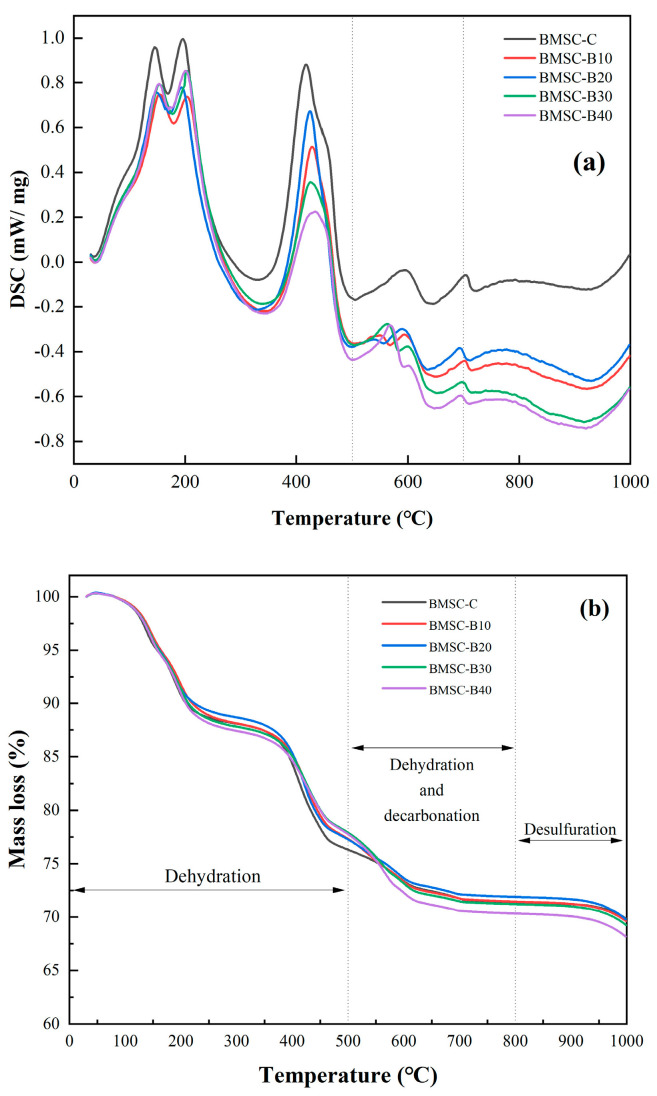
TG (**a**)–DSC (**b**) curves for the control and mixed samples with different mix proportions.

The TG-DSC curves of the control group and samples with different mix ratios are presented in Figure 9. The TG curve primarily depicts the decomposition process of the hydration product, namely the 5·1·7 phase. It can be observed from the TG curve that weight reduction in the range of BMSC-C to BMSC-B40 occurs in two stages. The initial stage, prior to 200 °C, corresponds to two absorption peaks in the DSC curve and is mainly attributed to the dehydration of the 5·1·7 phase into 5Mg(OH)_2_·MgSO_4_ (decomposition formulas shown in Equations (2) and (3)) [44,45]. Subsequently, a second stage of weight loss takes place within the temperature range of 400–600 °C, which also correlates with two absorption peaks on the DSC curve. With further increases in temperature, the continuous decomposition of 5Mg(OH)_2_·MgSO_4_ results in MgO, SO_3_, and H_2_O formation (decomposition formulas given by Equations (4) and (5)) [44,45].

On the TG curve, the weight reduction in BMSC-B40 surpasses that of BMSC-C, indicating that the incorporation of boron mud effectively inhibits the formation of 5·1·7 phase in the sample [46]. As the content of boron sludge increases, the degree of weight reduction becomes more pronounced. Simultaneously, after adding mineral admixtures, there is a decrease in the highest heat release rate for BMSC samples, which mitigates crystallization expansion stress induced by Mg(OH)_2_ and delays hydration kinetics in BMSC. Furthermore, at 600 °C, BMSC-B20 exhibits higher weight retention compared to BMSC-B10 due to superior embedding capability of fine particles from boron mud within microexpansion gaps in BMSC structure. This compensates for early volume instability caused by 5·1·7 phase and prevents further expansion resulting from additional MgO reaction [47]. This result does not differ significantly from other studies on calcined boron mud. The TG curves show similar weight loss patterns for BMSC-B10, BMSC-B20, BMSC-B30 and BMSC-B40; however, explanations regarding their strength differences will be provided through subsequent microstructural analysis.

Mg(OH)_2_·MgSO_4_·7H_2_O(s)→5Mg(OH)_2_·MgSO_4_·4H_2_O(s) + 3H_2_O (2)

5Mg(OH)_2_·MgSO_4_·4H_2_O(s)→5Mg(OH)_2_·MgSO4(s)·4H_2_O(g) (3)

5Mg(OH)_2_·MgSO_4_(s)→5MgO·MgSO_4_(s) + 5H_2_O(g) (4)

5MgO·MgSO_4_(s)→6MgO(s) + SO_3_(g) (5)

#### 3.1.4. FTIR Analysis

**Figure 10:**
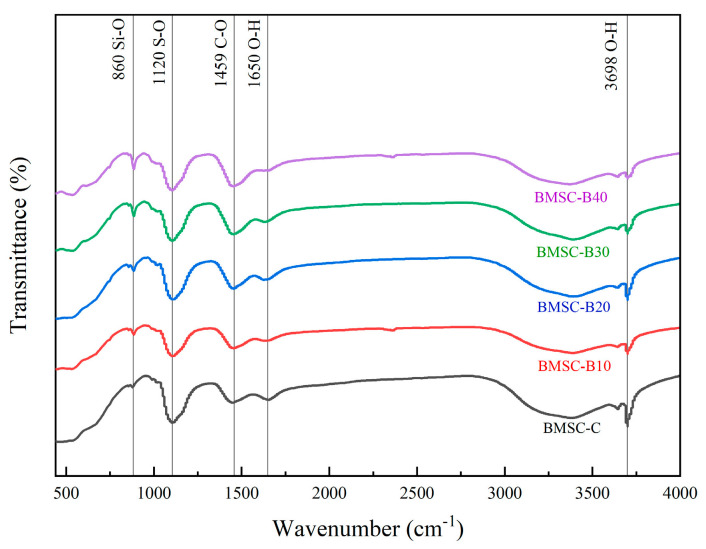
FTIR absorption spectra for control and mixed samples with different mix proportions.

The FTIR absorption spectra of the control group and samples with varying boron sludge content are presented in Figure 10. In each sample, the absorption bands at 1650 cm^−1^ and 3698 cm^−1^ correspond to the asymmetric stretching vibration and bending vibration peaks of O-H in crystallized water, respectively. The band at 860 cm^−1^ is attributed to the asymmetric stretching vibration peak of Si-O, and the band at 1120 cm^−1^ is due to the stretching vibration peak of S-O in sulfate ions. The absorption band at 1459 cm^−1^ corresponds to the stretching vibration peak of C-O in MgCO_3_ found in the BMSC samples. The 1120 cm^−1^ absorption band generated by BMSC-B10, BMSC-B20, and BMSC-B30 indicates the stretching vibration of S-O. This suggests that the addition of uncalcined boron mud does not significantly affect the formation of the 5·1·7 phase, as their spectral curves are similar to that of the control group BMSC-C, with only minor variations. However, the appearance of the absorption band at 1650 cm^−1^ can be attributed to the stretching vibration of O-H bonds. This change suggests that boron mud may reduce the free water content in the basic magnesium sulfate cement system, thus decreasing MgSO_4_ dissolution and potentially reducing the hydration heat during the cement hydration process [48]. Nevertheless, excessive boron sludge leads to a significant reduction in free water content within the basic magnesium sulfate system [49], which greatly affects MgSO_4_ dissolution and subsequently influences the formation of the 5·1·7 phase.

Overall, there are minimal differences observed in the flexural vibrations among BMSC-B10, BMSC-B20, BMSC-B30, and BMSC-B40 on the FTIR curves. Further explanations for their strength variations will be provided in subsequent microstructure analysis.

#### 3.1.5. SEM Analysis

**Figure 11:**
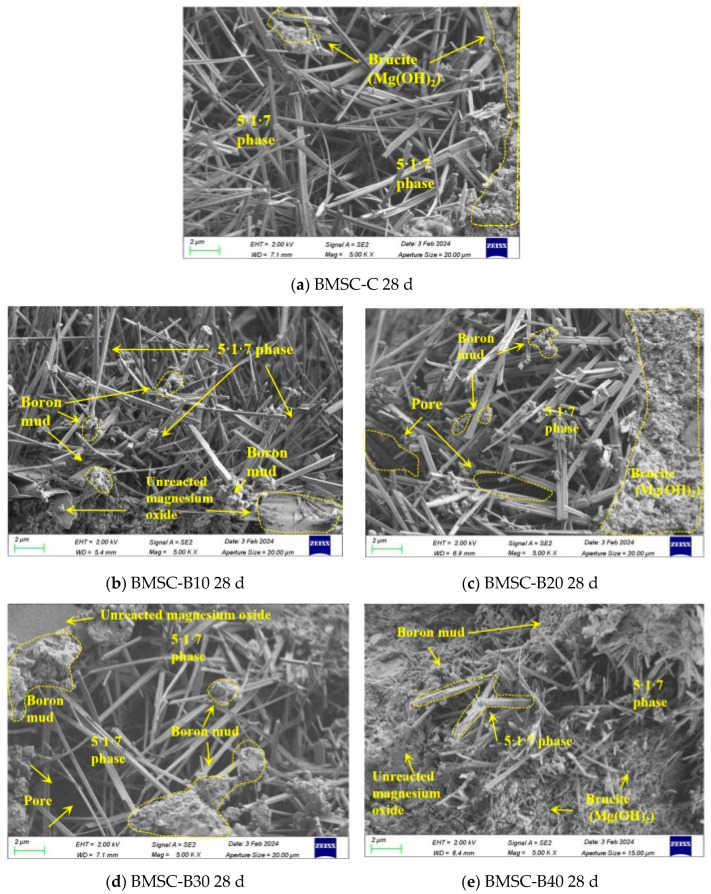
SEM for control group and BMSC samples with different mix proportions.

The SEM images in Figure 11a depict the microstructure of the conventional control group. It is evident from the figure that BMSC-C exhibits densely arranged whisker-shaped 5·1·7 phase structures, interwoven needle structures, and the overall stability of the BMSC structure.

The SEM image in Figure 11b illustrates BMSC-B10′s microstructure, which is generally similar to that of BMSC-C, except for the presence of a small amount of unreacted mineral impurities surrounding and within the 5·1·7 phase, primarily consisting of unreacted MgO particles and boron mud particles. The compressive strength at 28 days indicates that boron mud does not affect the strength of basic magnesium sulfate cement due to its high viscosity properties resembling clay [50]. According to Guo, J.B., et al.’s analysis, phase 5·1·7 undergoes complexation reaction [51]. By incorporating fine particles of boron mud into gaps between the 5·1·7 phase, its structure becomes more stable and ensures cement’s compressive strength. Furthermore, adding boron slime prevents volume expansion caused by Mg(OH)_2_ resulting from active magnesium oxide reacting with H_2_O [52], thereby ensuring bulk stability of the cement and leading to higher compressive strength at 28 days for BMSC-B10 compared to BMSC-C.

The SEM image of BMSC-B20 is presented in Figure 11c, revealing that the acicular structure of phase 5·1·7 remains unchanged compared to BMSC-C. Moreover, the compressive strength at 28 days is found to be similar to that of the control group. Notably, the presence of unreacted boron mud particles surrounding the 5·1·7 phase does not affect its formation. Additionally, Cao, J., et al. established that the 5·1·7 phase serves as the primary source of strength in the basic magnesium sulfate cement system [53]. These findings demonstrate that incorporating boron mud effectively fills any gaps within the cement matrix and prevents unreacted MgO from reacting with H_2_O to form Mg(OH)_2_. Furthermore, SEM analysis reveals an elongation in some needle-like whiskers and their interlacing arrangement further enhances structural stability.

The SEM image of BMSC-B30 is presented in Figure 11d, revealing the persistent presence of an acicular structure in phase 5·1·7. In terms of mechanical properties, although the mechanical strength at 28 days has decreased, the rate of decline is reduced compared to that observed in BMSC-B40. EDS analysis in Table 5 confirms a Mg to S atomic number ratio of 6:1 in BMSC-B30, providing evidence for the formation of a stable acicular crystalline phase within the gelled material.

The SEM image of BMSC-B40 is presented in Figure 11e, revealing a relatively shorter whisker length and increased surrounding impurities in the 5·1·7 phase. In terms of mechanical strength, the 28-day strength of BMSC-B40 exhibited a significant decrease compared to that of BMSC-B30 due to the larger specific surface area of boron sludge. Boron sludge reduces free water in the basic magnesium sulfate cement system and hampers the dissolution of MgSO_4_, thereby relatively reducing hydration heat during the process [20]. However, excessive boron sludge leads to an extreme reduction in free water content, greatly affecting MgSO_4_ dissolution and subsequently hindering the formation of 5·1·7 phase [54]. Therefore, after analyzing all BMSC specimens in this study under conditions with as much boron sludge as possible, it was found that the structure and mechanical properties were more stable for BMSC-B30 specimens. The decline rate in 28 days strength was relatively slow compared to that observed for BMSC-B40 specimens, achieving similar mechanical properties as those observed for control group samples. Microscopic analysis also revealed that a dosage of 30% boron sludge did not hinder the formation of the 5·1·7 crystalline phase.

**Figure 12:**
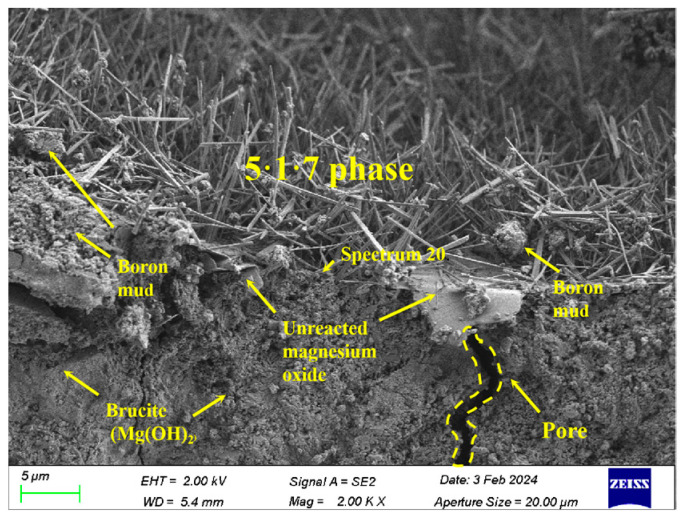
SEM image of BMSC-B30 sample.

Figure 12 shows the SEM diagram of the cement sample under optimal conditions at a magnification of 5 μm, which provides a better view of the microstructure compared to the 2 μm magnification in Figure 11. The SEM images of BMSC-B30 reveal needle-like crystal structures for the hydration products, while the surrounding flocculent particles correspond to unreacted mineral impurities. Compared to SEM figure in other studies on calcined boron mud, the structure of the 5·1·7 phase shows minimal differences. Each EDS analysis marked 10–20 target points to ensure the accuracy and reliability of the elemental composition data. The overall analysis indicates that boron mud particles do not significantly influence the formation of the 5·1·7 phase. Table 5 provides an EDS element distribution for the BMSC-B30 sample, calculated as an average from multiple analyses, adding to the robustness and credibility of the data. From this table, it can be inferred that the ratio of n(Mg):n(S):n(O) closely matches that of the 5·1·7 phase (7:1:24), suggesting a higher content of this needle bar morphology product. As a fundamental source contributing to sample strength, the staggered presence of the 5·1·7 phase better ensures volume stability in the cement.

## 4. Conclusions

This study investigated the feasibility of using uncalcined boron mud as a cementitious material for boron mud-based magnesium oxysulfate cement (BMSC). The effect of boron mud content on the compressive strength of BMSC was examined. Based on the results of this study, we can draw the following conclusions:

1. Uncalcined boron mud was selected to replace lightly burned magnesium oxide for the preparation of boron mud-based magnesium oxysulfate cement. TG and FTIR analyses indicated that the MgCO_3_ in the boron mud required calcination at 650 °C to 1000 °C to decompose, and the activity of magnesium oxide was only 13.76%. Additionally, Mg_2_SiO_4_ in boron mud required temperatures above 1700 °C to fully decompose. Therefore, uncalcined boron mud was preferred over pretreated boron mud.
2. A small amount of uncalcined boron mud was able to enhance the stability of the 5·1·7 phase structure. The fine particles of boron mud filled the small gaps within the BMSC, preventing unreacted free MgO from reacting with H_2_O to form Mg(OH)_2_. This ensured the volumetric stability of the 5·1·7 phase structure and the overall mechanical performance of the cement.
3. The XRD and SEM analyses of samples with 30% boron mud content revealed the optimal proportion. The compressive strength was 52.68 MPa at 7 days and 68.7 MPa at 28 days. Although there was some discrepancy in compressive strength compared to the control group, it fell within the expected range. The incorporation of 30% boron mud did not significantly affect the formation of the 5·1·7 crystalline phase, resulting in a relatively stable overall structure. This approach avoided potential environmental issues and significantly reduced the cost and energy consumption of MPC.

It was recommended to replace 30% of lightly burned MgO with boron mud to produce BMSC paste to manage the accumulated boron mud. This avoided potential environmental issues and significantly reduced the cost and energy consumption of BMSC. However, only boron mud from one region was selected in this study. The impact of boron mud from different regions on the performance of the composed BMSC requires further investigation. Future research could focus more on the chemical reaction characteristics of boron mud with the magnesium cement system and their impact on BMSC performance.

## Figures and Tables

**Table 1:** Chemical composition of boron mud.

Component	MgO	SiO_2_	Fe_2_O_3_	Al_2_O_3_	P_2_O_5_
Content (wt%)	73.51%	13.62%	10.2%	2.44%	0.23%

**Table 2:** Chemical composition of lightly burned magnesium oxide.

Component	MgO	CaO	SiO_2_	Fe_2_O_3_	Al_2_O_3_
Content (wt%)	83.3%	1.7%	6.34%	0.42%	0.18%

**Table 3:** Chemical composition of magnesium sulfate heptahydrate.

Component	MgSO_4_	MgCl_2_	NaCl	Na_2_SO_4_	H_2_O	Other
Content (wt%)	49.53%	0.6%	0.4%	0.5%	48.86%	0.2%

**Table 4:** The mix proportions of BMSC specimens.

Sample Types	Boron Mud (g)	MgO (g)	MgSO_4_ (g)	Citric Acid (g)	H_2_O (g)
BMSC-C	0 g	499 g	153.75 g	5 g	146.3 g
BMSC-B10	49.9 g	449.1 g	153.75 g	5 g	146.3 g
BMSC-B20	89.8 g	399.2 g	153.75 g	5 g	146.3 g
BMSC-B30	149.7 g	349.3 g	153.75 g	5 g	146.3 g
BMSC-B40	199.6 g	299.4 g	153.75 g	5 g	146.3 g

(Note: C—control group; B10–B40—boron mud replacement level ranging from 10% to 40%).

**Table 5:** Average distribution of elements in Spectrum BMSC-B30.

Element	Average Distribution in Spectrum BMSC-B30	
	Atomic Ratio/%	Molar Ratio/%
O	55.42	24.78
Mg	31.42	7.54
S	4.95	1

## Data Availability

The raw data supporting the conclusions of this article will be made available by the authors on request.
